# Chronic kidney disease alters lipid trafficking and inflammatory responses in macrophages: effects of liver X receptor agonism

**DOI:** 10.1186/s12882-018-0814-8

**Published:** 2018-01-27

**Authors:** Ryohei Kaseda, Yohei Tsuchida, Hai-Chun Yang, Patricia G. Yancey, Jianyong Zhong, Huan Tao, Aihua Bian, Agnes B. Fogo, Mac Rae F. Linton, Sergio Fazio, Talat Alp Ikizler, Valentina Kon

**Affiliations:** 1 Departments of Pediatrics, Vanderbilt University Medical Center, 1161 21st Avenue South, C-4204 Medical Center North, Nashville, TN 37232-2584 USA; 2 Pathology, Microbiology and Immunology, Vanderbilt University Medical Center, Nashville, TN USA; 3 Medicine, Vanderbilt University Medical Center, Nashville, TN USA; 4 Biostatistics, Vanderbilt University Medical Center, Nashville, TN USA; 5 Pharmacology, Vanderbilt University Medical Center, Nashville, TN USA; 6 Center for Preventive Cardiology, Knight Cardiovascular Institute, Oregon Health & Science University, Oregon, Portland USA

**Keywords:** HDL, CKD, Macrophages, Cholesterol efflux, LXR

## Abstract

**Background:**

Our aim was to evaluate lipid trafficking and inflammatory response of macrophages exposed to lipoproteins from subjects with moderate to severe chronic kidney disease (CKD), and to investigate the potential benefits of activating cellular cholesterol transporters via liver X receptor (LXR) agonism.

**Methods:**

LDL and HDL were isolated by sequential density gradient ultracentrifugation of plasma from patients with stage 3–4 CKD and individuals without kidney disease (HDL^CKD^ and HDL^Cont^, respectively). Uptake of LDL, cholesterol efflux to HDL, and cellular inflammatory responses were assessed in human THP-1 cells. HDL effects on inflammatory markers (MCP-1, TNF-α, IL-1β), Toll-like receptors-2 (TLR-2) and − 4 (TLR-4), ATP-binding cassette class A transporter (ABCA1), NF-κB, extracellular signal regulated protein kinases 1/2 (ERK1/2) were assessed by RT-PCR and western blot before and after in vitro treatment with an LXR agonist.

**Results:**

There was no difference in macrophage uptake of LDL isolated from CKD versus controls. By contrast, HD^CKD^ was significantly less effective than HDL^Cont^ in accepting cholesterol from cholesterol-enriched macrophages (median 20.8% [IQR 16.1–23.7] vs control (26.5% [IQR 19.6–28.5]; *p* = 0.008). LXR agonist upregulated ABCA1 expression and increased cholesterol efflux to HDL of both normal and CKD subjects, although the latter continued to show lower efflux capacity. HDL^CKD^ increased macrophage cytokine response (TNF-α, MCP-1, IL-1β, and NF-κB) versus HDL^Cont^. The heightened cytokine response to HDL^CKD^ was further amplified in cells treated with LXR agonist. The LXR-augmentation of inflammation was associated with increased TLR-2 and TLR-4 and ERK1/2.

**Conclusions:**

Moderate to severe impairment in kidney function promotes foam cell formation that reflects impairment in cholesterol acceptor function of HDL^CKD^. Activation of cellular cholesterol transporters by LXR agonism improves but does not normalize efflux to HDL^CKD^. However, LXR agonism actually increases the pro-inflammatory effects of HDL^CKD^ through activation of TLRs and ERK1/2 pathways.

## Background

Chronic kidney disease (CKD) has reached epidemic proportions, affecting 10–13% of the global population [1–3]. Cardiovascular disease (CVD) is responsible for most of the deaths in the CKD population. While CVD mortality is due to different causes, atherosclerotic CAD is consistently higher in CKD than in the general population [4, 5] and potentiation of CVD risk is seen even for modest impairment in kidney function, which constitutes the largest fraction of the CKD population [6–10]. The mechanisms by which CKD imparts the heightened CVD risk remain unclear, constraining development of risk-reduction interventions in this population. Lipid-lowering agents, primarily statins, are the therapeutic cornerstone to decrease CVD risk. However, even in the general population this intervention provides incomplete risk reduction, a shortcoming that becomes more evident among subjects with CKD.

The hallmark of atherosclerotic plaque is macrophage accumulation of cholesterol and transformation into foam cells, consequence of the unrelenting uptake of atherogenic lipoproteins, and of the failure to mobilize excess cholesterol to extracellular cholesterol acceptors, primarily HDL [11–13]. Epidemiologic studies have established that reduced HDL cholesterol (HDL-C) is associated with increased CVD; however, increasing HDL levels by drug therapies have not resulted in clinical benefits [14–16]. These observations have given rise to the concept of dysfunctional HDL to include impaired efflux capacity and pro-inflammatory properties. Importantly, recent human studies indicate that reduced HDL cholesterol efflux is an independent CVD risk factor [17–20], as capacity of HDL to stimulate cholesterol efflux from cultured macrophages predicted subclinical atherosclerosis and coronary artery disease in non-CKD populations. Consequently, conditions that impair cholesterol efflux or promote cellular uptake can accelerate atherosclerosis and CVD even in the absence of hypercholesterolemia, and may also foster resistance to treatment with lipid-lowering agents.

We, and others, have shown that patients with end stage renal disease (ESRD) requiring dialysis have dysfunctional HDL with reduced efflux and increased inflammation [21–30]. Less is known about the extent to which earlier stages of CKD affect HDL functionality. Although moderate CKD decreases HDL’s anti-inflammatory response, the impact on efflux capacity is less understood [22, 27, 29, 31–34]. It is also unclear whether, and which of the HDL functionalities can be modified. Increasing kidney function by renal transplantation can improve HDL anti-inflammatory/anti-oxidant function but not cholesterol handling [29]. There are no studies evaluating the utility of targeting macrophage cholesterol handling and inflammation that might constitute additional therapeutic targets beyond kidney transplantation or standard lipid lowering. Interestingly, we previously showed that uninephrectomy-induced amplification of murine atherosclerosis is linked to impaired cholesterol efflux reflecting repression of macrophage ATP-binding cassette transporter A1 (ABCA1) [35]. Liver X receptors (LXR) directly activate ABCA1, and experimental and clinical studies support potentially beneficial effects of LXR agonism to decrease foam cell formation and atherosclerosis in non-CKD settings [36, 37]. Therefore, we sought to evaluate both lipid handling and inflammatory response to lipoproteins of patients with moderate to severe (stage 3 and 4) CKD and assess the potential benefits of activating cellular cholesterol transporters with LXR agonism.

## Methods

### Study population

The study was approved by the Institutional Review Board at Vanderbilt University Medical Center (VUMC). Written informed consent was obtained from all participants. All subjects with CKD (*n* = 72) were followed at the Nephrology Clinic of VUMC. CKD stages 3–4 (GFR 30–59 mL/min/1.73 m^2^ and 15–29 mL/min/1.73 m^2^, respectively) were determined by CKD-EPI equation [38]. Healthy controls had estimated GFR > 60 mL/min/1.73 m^2^ and no proteinuria (*n* = 31). Patients with history of active connective tissue disease, acute infection within one month prior to the study, advanced liver disease, gastrointestinal dysfunction requiring parenteral nutrition, active malignancy, immunosuppressive drugs within one month prior to the study, history of myocardial infarction or cerebrovascular event within 3 months prior to the study were excluded. Some samples were insufficient and the number analyzed is shown in the Table and Results sections.

### Isolation of LDL and HDL fractions from blood

Blood samples were by collected by venipuncture into EDTA tubes, centrifuged at 1700 *g* for 15 min at 4oC and promptly processed. Five-hundred μl aliquots were stored at -80oC for biochemical determinations and NMR lipoprotein analysis, while the rest of the plasma was further processed for isolation of HDL or LDL (CKD, *n* = 63 and Controls, *n* = 25). The HDL fractions (d = 1.063 to 1.21 g/ml) and LDL fractions (d = 1.019 to 1.063 g/ml) were isolated from fresh plasma of each subject by density gradient ultracentrifugation after adjustment with potassium bromide [39]. HDL was frozen at -80 °C and thawed only once which we and others have shown as having minimal effect on functionality [18, 21, 40]. LDL was used immediately after isolation.

Plasma levels of total cholesterol, triglycerides and HDL were measured enzymatically (Cliniqa, CA). High-sensitivity C-reactive protein (hsCRP) was measured using a high sensitivity immunoturbidimetric assay as previously described (Roche Modular System, Indianapolis, IN) [41]. HDL particle concentration and particle size of lipoprotein was evaluated from 500 μl plasma by LIPOSCIENCE (Raleigh, NC) using their proprietary NMR methodology.

### Macrophage uptake of LDL and cholesterol efflux from lipid-loaded macrophages to HDL

THP-1 cells were plated and differentiated into macrophages by RPMI 1670 containing 10% fetal bovine serum and 50 ng/ml phorbol 12-myristate 13-acetate [21]. The cells then incubated with low density lipoprotein (LDL) (100 μg/ml) for 24 h. Cholesterol content was measured by gas chromatography [39, 42] and cholesterol uptake determined as the percent at baseline versus after incubation with LDL [43]. Cell protein content was measured by bicinchoninic acid assay. Efflux assessment followed many of the steps described for uptake including cellular differentiation, which was followed by cholesterol enrichment with acetylated LDL (100 μg/ml, Intracel, Frederick, MA) and exposure to HDL (50 μg/ml) and LPS (50 ng/ml) for 24 h. Cellular cholesterol content was measured by gas chromatography and efflux determined as the percent cellular cholesterol at baseline versus post HDL exposure as described [39, 42].

### Macrophage inflammatory cytokine response

Macrophage expression of cytokines, inflammatory markers, transporters and signaling molecules were assessed by RT-PCR and western blotting. HDL modulation of the inflammatory effect was measured using the established cytokine response in LPS–activated macrophages [21]. THP-1 cells were plated and differentiated into macrophages. Total RNA was extracted from cells with Trizol reagent (Life Technologies, Carlsbad, CA). Quantification of human interleukin (IL)-1β, monocyte chemoattractant protein-1 (MCP-1), tumor necrosis factor α (TNFα), and endogenous control human Euk 18S rRNA levels was performed by real-time reverse transcriptase polymerase chain reaction (PCR) using CFX96™ Real-Time System (BIO-RAD, Hercules, CA). Probes for IL-1β (Hs99999029_m1), MCP-1 (Hs00234140_m1), TNFα (Hs99999043_m1), ABCA1(Hs01059122_m1), Toll like receptor (TLR)2(Hs01872448_s1), TLR4(Hs00152939_m1) and 18S rRNA were obtained from Applied Biosystems (Foster City, CA). For western blot assessment, macrophages exposed to HDL and LPS for 4 h were lysed with RIPA buffer from Boston Bio Products (Ashland, MA). Equal amounts of protein were separated on 4–12% Nu PAGE Bis-tris gel and transferred onto nitrocellulose membranes immunostained with primary antibodies against phosphorylated p44/42 MAPK from Cell Signaling Technology (Danvers, MA) and subsequently with HRP-conjugated second antibody. Blots were visualized using Western Lightning Plus-ECL from Perkin Elmer (Waltham, MA) and developed on film. After incubation with Restore Western Blot Stripping Buffer from Thermo (Rockford, IL), the membranes were immunostained with primary antibody for total p44/42 MAPK from Cell Signaling Technology. To quantify protein expression, western blots were analyzed by ImageJ. NF-κB activity was determined as previously described [44]. Briefly, 10 μg nuclear extract from each of the groups was incubated with 1 μL of 50 nM NF-κB oligonucleotide (LI-COR Biotechnology). for 20 min, gel assay performed in 5% TBE native acrylamide gel. The signals were captured on Odyssey scan system (Li-COR Biotechnology). In studies to activate ABC transporter, liver X receptor (LXR) agonist T0901317 (1 μM, SIGMA) was added.

#### Statistics

Results are expressed as a mean of triplicate assays. Descriptive statistics are presented as frequencies and percentages for categorical variables and median (interquartile range [IQR]) for continuous variables. Demographic and clinical factors were compared between control and patients with CKD using Wilcoxon test for continuous variables and Pearson Chi-square test for categorical variables. Spearman correlation was used to assess the correlation between age and efflux in controls and CKD patients. Separate proportional odds models were used to examine the association between efflux or cytokines and group with adjustment for age. Statistical analyses were performed using R version 2.10.0. A 2-sided significance level of 5% was deemed statistically significant.

## Results

### Characteristics of study subjects

Demographic, clinical and biochemical characteristics of all participants are summarized in Table 1. The CKD group was older although sex and race were not different. There were no statistically significant differences between CKD and controls in levels of plasma total cholesterol, LDL, HDL or the size of the HDL particles. As expected, CKD patients had significantly elevated plasma triglycerides and higher hsCRP levels than controls.

**Table 1:** Characteristics of Study Subjects

	Control (*n* = 31)	CKD (*n* = 72)	*P* Value
Age (year)	49.9 ± 10.6	61.9 ± 13.5	*P* < 0.05
Gender (male/female)	11/20	40/32	NS
Race (AA/Caucasian/Other)	1/29/1	15/56/1	NS
eGFR (ml/min/1.73m^2^)	≧60	33.7 ± 11.3	*P* < 0.05
hsCRP (mg/dl)	3.83 ± 5.47	8.22 ± 9.90	*P* < 0.05
Total cholesterol (mg/dl)	158 ± 37	162 ± 44	NS
TG (mg/dl)	98 ± 43	161 ± 97	P < 0.05
LDL (mg/dl)	92 ± 33	88 ± 31	NS
HDL size (nm)	9.12 ± 0.46	9.07 ± 0.51	NS
HDL-C (mg/dl)	44.5 ± 8.4	40.9 ± 12.3	NS
HDL particles (μmol/L)	30.1 ± 5.7	28.5 ± 6.2	NS
• Large HDL particles (μmol/L)	5.3 ± 2.7	4.8 ± 3.1	NS
• Medium HDL particles (μmol/L)	9.4 ± 5.7	7.5 ± 5.3	NS
• Small HDL particles (μmol/L)	15.4 ± 6.2	16.1 ± 6.2	NS
apoAI1 (mg/dl)	125 ± 16	123 ± 24	NS

*AA* African American, triglyceride, *HDL* high density lipoprotein, *LDL* low density lipoprotein, *hsCRP* high-sensitivity C reactive protein. hsCRP and all lipid and lipoprotein assays were done in Controls (*n* = 18) and CKD (*n* = 53)

### Macrophage uptake of LDL

Since foam cell formation reflects an imbalance between lipoprotein uptake and cholesterol efflux, we evaluated the macrophage cholesterol-loading capacity with the LDL fractions isolated from individuals with normal kidney function (73.1% [67.5, 75.6], *n* = 5) and those with CKD (64.9% [59.4, 70.4], n = 5, *p* = 0.63). LDL isolated from CKD caused a similar degree of cholesterol accumulation in THP-1 macrophages as did LDL isolated from controls.

### Efflux capacity of HDL

**Fig. 1:**
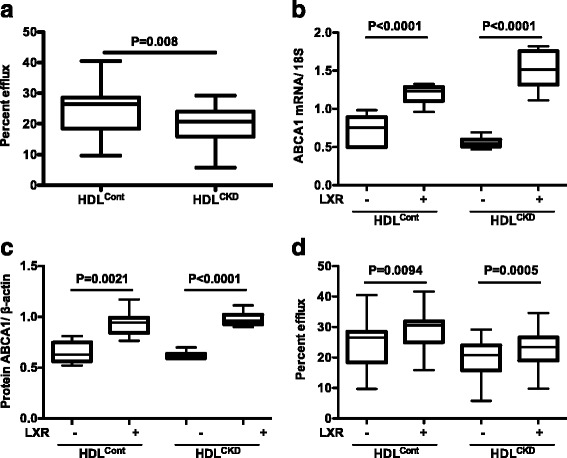
Efflux from lipid loaded THP-1 macrophages to HDL isolated from patients with chronic kidney disease (CKD) and controls with normal kidney function (Control) (**a**). ABCA1 mRNA and protein in THP-1 macrophages with/without LXR agonist (**b** and **c**). Cholesterol efflux from THP-1 macrophages with/without activation of ABCA1 transporters by LXR agonist (**d**)

In contrast to cellular uptake of LDL isolated from CKD (*n* = 58) and controls (*n* = 20), cholesterol acceptor function from cholesterol loaded THP-1 cells was significantly lower for HDL^CKD^ versus HDL^Cont^ (median 20.8%, IQR 16.1–23.7, vs 26.5%, IQR 19.6–28.5, *n* = 49, 20, respectively, *p* = 0.01) (Fig. 1a). Efflux was not significantly correlated with age in the CKD group (spearman rho = 0.02, *p* = 0.90) or in controls (spearman rho = 0.37, *p* = 0.11) [21]. After adjustment for age, there was no significant difference between the CKD and control groups (*p* = 0.42).

To assess whether activation of cellular transporters affect the acceptor capacity of HDL, we exposed THP-1 cells to an LXR agonist (T0901317), which significantly increased gene and protein expression of ABCA1 in cells exposed to HDL^CKD^ and HDL^Cont^ (Fig. 1b, c). Up-regulation in cellular ABCA1 significantly increased cholesterol efflux to HDL^Cont^. Although LXR agonism also increased cholesterol efflux with exposure to HDL^CKD^, efflux to HDL^CKD^ remained significantly lower than HDL^Cont^ (*P* < 0.005) (Fig. 1d). The difference in efflux in LXR-exposed cells persisted between CKD and control groups after adjustment for age (*p* = 0.01).

### Anti-inflammatory capacity of HDL

**Fig. 2:**
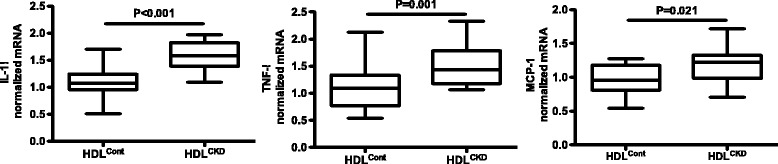
Cytokine expression by THP-1 macrophages in response to HDL^Control^ and HDL^CKD^ with expression of interleukin-1β (IL-1β), and tumor necrosis factor (TNF)-α and monocyte chemoattractant protein (MCP-1) measured by real-time PCR

**Fig. 3:**
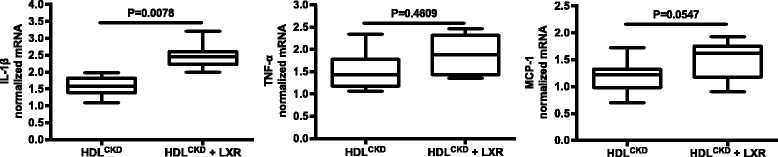
Cytokine expression of LXR-treated THP-1 macrophages to HDL^CKD^ with expression of interleukin-1β (IL-1ββ, and tumor necrosis factor (TNF)-α and monocyte chemoattractant protein (MCP-1) measured by real-time PCR

We also investigated the cellular inflammatory response elicited by HDL from the same subjects in whom we assessed cholesterol handling. HDL^CKD^ caused a greater cytokine response in THP-1 cells compared with that observed in cells treated with HDL^Cont^ (Fig. 2). After adjustment for age, there were statistically significant differences between CKD and controls for the proinflammatory cytokines (IL1β, *p* < 0.001; TNFα, *p* = 0.04; MCP-1, *p* = 0.06). Further, while exposure of LXR agonist-treated cells to HDL^Cont^ did not significantly affect the TNFα or MCP-1 cytokine response (TNFα, 1.09 [0.79, 1.32] vs 1.24 [1.21,1.61] with LXR, *p* = 0.94, and MCP-1, 0.96 [0.81, 1.17] vs 1.21 [0.77, 1.33] with LXR, p = 0.94), exposing LXR-treated cells to HDL^CKD^ potentiated the inflammatory response, further increasing cellular expression of IL-1β (*p* < 0.01) and MCP-1 (*p* = 0.055) (Fig. 3).

**Fig. 4:**
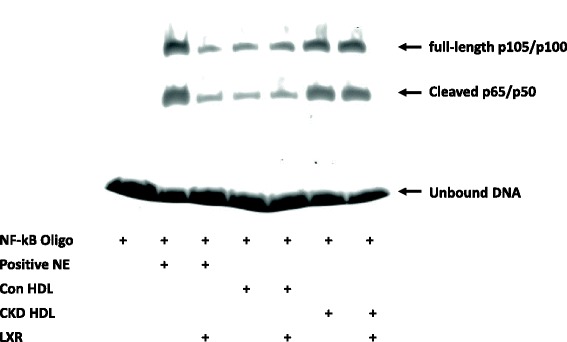
Electrophoretic mobility-shift assay (EMSA) of NF-κB activity in macrophages with/without LXR agonist exposed to HDL isolated from patients with chronic kidney disease (CKD) and controls with normal kidney function (Control)

**Fig. 5:**
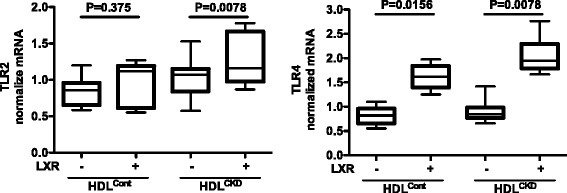
Toll-like receptor-2 (TLR-2) and − 4 (TLR-4) responses of THP-1 macrophages to HDL^Control^, HDL^CKD^ with/without LXR-treatment

**Fig. 6:**
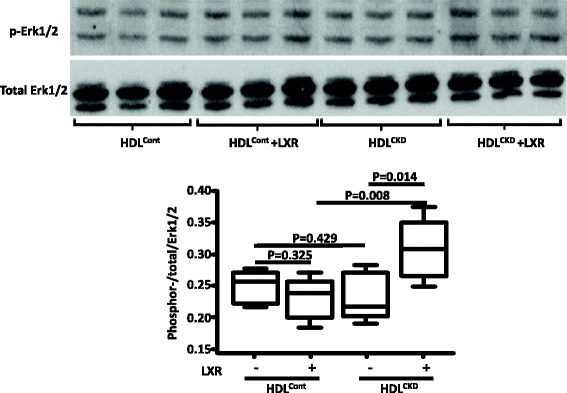
ERK1/2 in THP-1 macrophages exposed to HDL^Control^, HDL^CKD^ with/without LXR-treatment

We next examined the possible pathways involved in the enhanced inflammatory response of LXR-treated cells to HDL^CKD^. The amplification was not related to a differential effect on NF-κB. Whereas cells exposed to HDL^CKD^ had greater abundance of NF-κB than cells exposed to HDL^Cont^, there was little difference between LXR-treated and untreated cells (Fig. 4). By contrast, while LXR agonist had little effect on TLR2 expression in cells exposed to HDL^Cont^, there was a significant increase in TLR2 expression in the cells exposed to HDL^CKD^ (Fig. 5). Treatment with LXR agonist also increased TLR4 expression in cells exposed to either HDL^Cont^ or HDL^CKD^, a result compatible with TLR4 being a ligand for LPS, which we used in our experimental procedure (Fig. 5). Since ERK1/2, play critical roles in production of proinflammatory cytokines, we also examined this pathway in the HDL^CKD^–induced cytokine potentiation in LXR treated cells. There was no difference in ERK1/2 activation between cells exposed to HDL^Cont^ or HDL^CKD^. However, in cells treated with LXR agonist, only HDL^CKD^ but not HDL^Cont^ increased ERK1/2 activation (Fig. 6).

## Discussion

Our data indicate that HDL isolated from patients with moderate to severe CKD has impaired capacity to elicit cholesterol efflux from lipid-loaded macrophages and potentiates inflammation, compared with HDL of individuals with normal kidney function. Both cholesterol handling and regulation of vascular inflammation represent potentially atheroprotective functions of HDL that may be influenced by pharmacologic intervention. We show that upregulation of ABCA1 transporters with LXR agonism significantly increases cellular cholesterol efflux both to normal HDL and to the dysfunctional HDL from CKD patients. However, and unexpectedly, LXR activation also amplifies the already heightened cellular inflammatory cytokine response to HDL^CKD^ by mechanisms linked to activation of TLRs and the ERK1/2 pathway.

These results have important implications. First, our data show that even moderate CKD modifies HDL function and promotes cellular cholesterol accumulation. Interestingly, the lipid accumulation does not reflect greater uptake of LDL. Although macrophage uptake of unmodified LDL is small, CKD is well recognized to cause LDL oxidation which would augment cellular cholesterol loading via scavenger receptor uptake [45]. However, we did not see increased uptake and instead observed significantly impaired cholesterol efflux, results that echo previous findings by us and others in patients with ESRD on dialysis [21, 23, 24, 27, 29]. The results also complement findings by Shroff et al. who reported a graded change in cholesterol efflux capacity in children with CKD stage 2–5 [29]. By contrast, a recent study reported no difference in cholesterol efflux capacity of HDL from normal subjects and individuals with various degrees of kidney impairment (pre-dialysis, maintenance hemodialysis and transplant recipients). However, the HDL concentration used in the assay was only 20% of that commonly applied in efflux assays and may have been insufficient to effectively elicit cholesterol movement from lipid-laden cells [46]. Taken together, our findings support the hypothesis that even moderate kidney impairment causes HDL dysfunction, including reduced capacity for cholesterol efflux. These observations have particular importance since moderate to severe degree of CKD comprise > 50% of all patients with kidney disease and because efflux capacity has been shown to be a marker for atherosclerosis and risk of cardiovascular events in individuals without kidney damage, although not patients requiring dialysis [17, 18, 47]. Implications of these findings are further heightened by the fact that standard lipid-lowering therapies reduce the CVD risk by only 20%. Thus, there is the potential of sizable additional residual CV risk reduction in the high risk CKD population using therapies targeted at this derangement [48].

Our data suggest that age might have some impact on HDL’s efflux capacity, observations that complement the previously described effects of aging on HDL function [49]. Specifically, adjustment for age eliminated the statistically significant difference in efflux capacity between the CKD and control groups. Interestingly, there were no differences in efflux capacity between CKD and controls in individuals who were less than 50 years old. It is interesting that cellular activation of LXR also increased cholesterol efflux to both HDL^Cont^ and HDL^CKD^, the statistically significant difference persisting after adjustment for age. These data suggest the possibility that, even in the face of dysfunctional HDL, improving cellular transporter activity can increase cholesterol export, which would predict a reduction in foam cells in the arterial wall. Our finding indicate that this may be beneficial to CKD patients regardless of age. Such therapeutic possibility is supported by experimental observations that synthetic LXR agonists can decrease atherosclerosis in animal models through mechanisms that include macrophage efflux and increased reverse cholesterol transport [37, 50].

In addition to modulating cholesterol transport, LXR activators have been shown to have anti-inflammatory effects that may be critically important in their antiatherogenic effects [36]. Therefore, we also assessed the effects of LXR agonism on the cellular inflammatory response using the same HDL samples applied to the efflux assay. Our results show macrophage expression of IL-1β, IL-6, and TNF-α is greater in response to HDL^CKD^ than HDL^Cont^. Furthermore, LXR-activated cells exposed to HDL^CKD^ showed unexpected potentiation in the inflammatory response (Fig. 3). These results suggest that while HDL^CKD^ is abnormal in terms of both efflux and inflammation, the abnormalities are not synchronized and therapeutic modalities may have variable effects on distinct HDL actions.

Interestingly, a recent study reported that treatment of LDLR^−/−^ mice with the LXR activator T0901317, substantially reduced the extent of atherosclerotic lesions, independent of the macrophage ABCA1/G1 cholesterol efflux pathways [36]. Instead, the beneficial effects of LXR activation reflected the anti-inflammatory actions of LXR thought to be mediated through transrepression mechanisms involving the small ubiquitin-like modifier (SUMO)-ylation of LXR, that targets promoters of NF-κB target genes and results in repression of inflammatory genes downstream of NF-κB [51]. Our study shows that compared to macrophages exposed to normal HDL, cells exposed to HDL^CKD^ have dramatically increased activity of NF-κB (Fig. 4), central role of NF-κB in regulation of inflammatory processes when renal function is reduced [52]. Notably, however, activation of LXR did not affect expression of NF-κB, suggesting that increased cytokine response in this context does not involve the NF-κB-dependent transrepression pathway. Instead, LXR agonism was associated with direct effects on TLR2 and TLR4. While TLR2 and TLR4 can activate the same signaling pathway, such as JNK, the ERK1/2 pathway is primarily activated by TLR2 [53]. Our data show that both HDL^Cont^ and HDL^CKD^ activate TLR4, whereas only HDL^CKD^ activates both TLR4 and TLR2. Moreover, TLR2 activation was associated with phosphorylation of ERK1/2 in response to HDL^CKD^. Thus, whereas both TLR4 and TLR2 can stimulate a cytokine response to HDL^Cont^ and HDL^CKD^, the exaggerated response to HDL^CKD^ is more closely linked to TLR2 than TLR4. Our findings that TLRs are involved in HDL^CKD^-augmented inflammatory response complements previous reports that TLR2 has a critical role in impaired endothelial protection and anti-inflammatory capacity of HDL isolated from adults and children with stage 2–4 CKD [33]. It is interesting that in human macrophages pretreatment with LXR agonists for 24 h significantly reduced the inflammatory response induced by LPS whereas longer exposures paradoxically enhanced the inflammatory response [50]. Further, the increased cytokine production involved TLR4 [54, 55], as well as cytokine production through pathways driven by TLR2 [55]. The LXR augmentation of TLR-induced cytokine secretion has been proposed as the molecular pathway responsible for synovial inflammation in rheumatoid arthritis [55]. Similarly, it is possible that in the CKD setting, LXR agonism produces undesirable effects by activating TLRs and further potentiating cytokine production.

## Conclusions

Patients with moderate to severe CKD have dysfunctional HDL, which causes impaired cholesterol acceptor function and enhanced inflammatory response in cultured macrophages. Improved cholesterol efflux to HDL^CKD^ was achieved by activation of the cellular ABCA1 transporter via LXR. However, our results also show that the potential benefits of LXR-agonism to improve HDL^CKD^ cholesterol acceptor capacity are possibly offset by its potentiation of the cellular inflammatory cytokine response, which was mediated by activation of TLR2 and 4 and ERK1/2 signaling pathway.

## Abbreviations

ABCA1: ATP-binding cassette class A transporter
CKD: Chronic kidney disease
CVD: CARDIOVASCULAR disease
ERK1/2: Extracellular signal regulated protein kinases 1/2
ESRD: End stage renal disease
GFR: Glomerular filtration rate
HDL: High-density lipoprotein
HDL-C: HDL cholesterol
hsCRP: High-sensitivity C-reactive protein
IL-1β: Interleukin-1β
IQR: Interquartile range
JNK: c-Jun N-terminal kinases
LDL: Low-density lipoprotein
LPS: Lipopolysaccharides.
LXR: Liver X receptor
MAPK: Mitogen-activated protein kinases
MCP-1: Monocyte chemoattractant protein-1
NF-κB: Nuclear factor kappa-light-chain-enhancer of activated B cells
RT-PCR: Real-time reverse transcriptase polymerase chain reaction
TLR-2: Toll-like receptors-2
TLR-4: Toll-like receptors-4
TNF-α: Tumor necrosis factor α
